# Two opposite abilities of the infectious bronchitis virus helicase Nsp13: separating the duplex and promoting the annealing of single-stranded nucleic acid

**DOI:** 10.3389/fvets.2025.1560586

**Published:** 2025-03-17

**Authors:** Chengcheng Wu, Lihan Tao, Haiqin Li, Cong Huang, Yanbing Zeng

**Affiliations:** ^1^Institute of Animal Husbandry and Veterinary Medicine, Jiangxi Academy of Agricultural Sciences, Nanchang, China; ^2^College of Animal Science and Technology, Jiangxi Agricultural University, Nanchang, China

**Keywords:** coronavirus, helicase, unwinding, annealing, replication mechanism

## Abstract

Genome replication is a key step in the coronavirus life cycle and requires the involvement of a range of virally encoded non-structural proteins. The non-structural protein 13 (Nsp13) of coronaviruses is a highly conserved helicase and is considered an ideal drug target. However, the activity characteristics of the helicase Nsp13 of the infectious bronchitis virus (IBV) remain unclear. In this study, we expressed and biochemically characterized the purified recombinant IBV Nsp13 and found that IBV Nsp13 was able to unwind duplex substrates in a 5′-to-3′ direction by using the energy from the hydrolysis of all nucleotide triphosphate (NTP) and deoxyribonucleoside triphosphate (dNTP). We also explored the substrate selectivity and influencing factors of the unwinding activity of IBV Nsp13. The nucleic acid continuity of the loading strand was essential for Nsp13 to unwind duplex substrates. In addition, we first demonstrated that IBV helicase Nsp13 also had an annealing activity to promote two single-stranded nucleic acids to form a double-stranded nucleic acid. Biochemical analysis of the unwinding and annealing activities of IBV Nsp13 was helpful for deeply revealing the replication mechanism of coronavirus and the development of antiviral drugs.

## 1 Introduction

Coronaviruses are major pathogens that infect humans and animals, causing serious public health concerns and large economic losses. Coronaviruses belong to the family *Coronaviridae* and are taxonomically classified into four genera based on their serotype and genomic characteristics: *Alphacoronavirus*, *Betacoronavirus*, *Gammacoronavirus,* and *Deltacoronavirus* (1). Coronaviruses of *Betacoronavirus* are the most dangerous to humans, and severe acute respiratory syndrome coronavirus-2 (SARS-CoV-2) has been responsible for the COVID-19 pandemic since 2019 (2). Porcine deltacoronavirus (PDCoV) from *Deltacoronavirus* was detected in the blood of three children in Haiti in 2021 and also posed a risk of infecting humans (3).

The avian infectious bronchitis virus (IBV) is an enveloped single-stranded positive-sense RNA virus that belongs to the genus *Gammacoronavirus*. IB caused by IBV is an acute and highly contagious infectious disease in chickens with a significant impact on the global poultry industry (4). IBV can infect chickens of all ages and types, leading to respiratory distress, nephritis, salpingitis, and egg production decline (5). The IBV genome is approximately 27.6 kb and encodes the polyproteins 1a (pp1a) and 1ab (pp1ab), which are proteolytically cleaved by two virus-encoded proteinases, papain-like protease (PLpro) and 3-chymotrypsin-like protease (3CLpro), into 15 mature non-structural proteins (Nsp2 ~ Nsp16) (6). Compared to other coronaviruses, Nsp1 is absent in IBV, but Nsp2 is considerably larger.

Helicases are ubiquitous molecular motor proteins that harness the energy derived from ATP hydrolysis to catalyze the unwinding of energetically stable double-stranded (ds) nucleic acids into two single-stranded (ss) nucleic acids and thus play important roles in nearly all aspects of nucleic acid metabolism, including replication, repair, recombination, and transcription (7). Helicases were divided into six superfamilies (SFs) by the comparative analysis of their structure and function. The non-structural protein Nsp13 of coronavirus is a highly conserved helicase that belongs to SF1, which is essential for virus replication and thus a potential target for antiviral drug design (8). For instance, some bismuth-based compounds inhibited SARS-CoV-2 Nsp13 activities, exhibited low cytotoxicity, and suppressed SARS-CoV-2 replication (9, 10). The Nsp13 contains a zinc-binding domain, a Stalk domain, a 1B domain and a core helicase domain; the structure coordination of domains is important for the translocation and unwinding of helicase (11). Previous studies showed that Nsp13 of SARS-CoV and SARS-CoV-2 localized on the endoplasmic reticulum membrane, hydrolyzed ATP, and unwound dsRNA and dsDNA in a 5′ to 3′ orientation (12), and the helicase Nsp13 of MERS-CoV and that of PEDV have similar functions (13, 14). An arginine-to-proline mutation at amino acid position 132 of the helicase Nsp13 is lethal to IBV infectivity in Vero cells (15). However, the biochemical characterization of IBV helicase Nsp13 remains largely unknown.

In this study, purified IBV Nsp13 was obtained, and its catalytic functions were biochemically characterized. Partial duplex RNA and DNA were used as model and control substrates, and we found that IBV Nsp13 had strong ATPase and helicase activities. All NTP and dNTP could be hydrolyzed to provide energy for the unwinding of duplex substrates in a unidirectional fashion. Moreover, species of divalent metal ions affect the activity of Nsp13, and the nucleic acid continuity of the loading strand (the strand contained a 5′-overhang, and the helicase was presumed to translocate) of duplex substrates was indispensable for the unwinding reaction. Furthermore, we found for the first time that IBV Nsp13 could catalyze the annealing of two single strands to produce a double strand in the absence of ATP; this function was opposed to unwinding and might also play an equally important part in the process of viral replication.

## 2 Materials and methods

### 2.1 Protein expression and identification

IBV helicase gene *Nsp13* was downloaded from GenBank (accession no. MN548289.1) and synthesized by the biotech company (General Biol, Chuzhou, China). Thereafter, the DNA fragment was oriented and inserted into the vector pET-28a to generate the prokaryotic expression plasmid pET-28a-Nsp13. The recombinant plasmid was verified by DNA sequencing and transformed into *Escherichia coli* BL21 (Takara, Japan). It was grown in LB medium with Kanamycin at 37°C until the optical density at 600 nm (OD600) reached 0.6–0.8. The protein was induced to be expressed with 0.2 mM isopropyl-beta-D-thiogalactopyranoside (IPTG) overnight at 18°C, and the shaker rotational speed was 120 r/min. Cells were harvested by centrifugation at 14000 r/min and resuspended in Buffer A (500 mM NaCl, 1 mM phenylmethylsulfonyl fluoride (PMSF), and 25 mM Tris–HCl, pH = 8.0). Then, cells were lysed by sonication (Scientz, Ningbo, China), followed by centrifugation at 20000 r/min for 30 min. The supernatants were filtered through a 0.45 μm filter (Millipore, USA) and eluted with a linear gradient concentration of imidazole from 20 mM to 500 mM through Ni-affinity columns. The eluates were centrifuged in an ultrafiltration centrifugal tube (Millipore, USA), and the buffers were replaced with Buffer B (200 mM NaCl and 200 mM Tris–HCl, pH = 8.0). All the above steps were implemented at 4°C. The purity and identity of proteins were evaluated by Coomassie brilliant blue R250 staining and Western blotting analysis with anti-His antibody, respectively. Protein samples were divided into 20 μL/vial, quickly frozen in liquid nitrogen, and stored at −80°C. The concentration of the purified IBV Nsp13 was measured using the Bradford protein assay kit (Beyotime, Shanghai, China).

### 2.2 Preparation of nucleic acid substrates

5-Carboxyfluorescein (5-FAM)-labeled RNA and DNA were synthesized by the biotech company (Tsingke, Beijing, China). The efficiency of all the labeling reactions was proved higher than 80% according to the RNA extinction coefficient and fluorophores measured by UV–vis spectroscopy (Shimadzu, Japan). The purification of RNA was implemented by native PAGE. Supplementary Table S1 lists the oligonucleotide sequences used to generate duplex substrates in this study. The dsRNA with 5′-overhang, 3′-overhang, and blunt-end was generated by the annealing reaction of FAM-labeled RNA with unlabeled RNA at a ratio of 1:1 in the buffer (100 mM NaCl and 50 mM Tris–HCl, pH =7.5). The mixture was heated at 95°C for 5 min and then cooled down slowly to room temperature. The annealing procedures for ssDNA for dsDNA generation were the same as for dsRNA. Annealing products were diluted into required concentrations with diethyl pyrocarbonate (DEPC) water and stored at −20°C. Furthermore, to prevent RNA degradation, we implemented multiple safeguards, including (1) using RNase-free tips and centrifuge tubes throughout the experimental procedures, (2) preparing all reagents with DEPC-treated water, and (3) incorporating RNase inhibitors into the reaction system.

### 2.3 ATPase assay of IBV helicase Nsp13

Kinase-lumi max luminescent kinase assay kit (Beyotime, Shanghai, China) was used to detect the ATPase activity of IBV Nsp13 by chemiluminescence determination of the remaining amount of ATP in the solution. In brief, Nsp13 in reaction buffer (30 mM Tris–HCl, 3 mM MgCl_2_, 2 mM dithiothreitol (DTT), and 5 μM ATP, pH = 7.5) was added to a 96-well white plate (LabSelect, China) with deionized water to a total volume of 50 μL. The plate was then incubated in a 25°C incubator for 30 min. At the end of the reaction, 50 μL of kinase-lumi reagent was added to the reaction mixture. After mixing and incubation at 25°C for 10 min, the luminescence of each well was measured by a SpectraMax iD5 multifunctional microplate reader (Molecular Devices, USA). The data are represented as mean ± standard deviation to visualize measurement variability. All experiments were repeated three times independently.

### 2.4 Unwinding assay of duplex substrates

An electrophoretic mobility shift assay (EMSA) was conducted to evaluate the unwinding activity of IBV helicase Nsp13. The unwinding reaction was performed in reaction buffer (30 mM Tris–HCl, 0.1 mg/mL of BSA, 1% glycerol, 2 mM DTT, 1 unit/μL of RNase inhibitor, 50 nM trapRNA, 4 nM dsRNA, and 3 mM MgCl_2_) for the indicated time. Before the reaction, Nsp13 and ATP were preincubated separately at 37°C for 5 min, and the unwinding reaction was initiated by adding a certain concentration of ATP. Reactions were quenched by adding an equal volume of stop buffer (50 mM EDTA·Na_2_·2H_2_O, 1% SDS, and 10% glycerol). The unwinding reaction of dsDNA was similar to that of dsRNA. Reaction products were resolved using 12% native PAGE and then run in Tris-borate–EDTA (TBE) buffer (89 mM Tris-borate and 2 mM EDTA·Na_2_·2H_2_O) at room temperature with 150 V for 2 h. Images were obtained by scanning gels with ImageQuant500 (Cytiva, USA) or Amersham Typhoon RGB laser scanning imager (Cytiva, USA). The band intensities of the reaction products were extracted by ImageJ software, and the data points were fitted to a single-exponential equation:


$$F\left(t\right)=A*\left(1-\exp\left(-k*t\right)\right)$$

*F(t)* is the fraction unwound at time *t*, *A* is the reaction amplitude (defined as the final fraction of the unwound duplex), and *k* is the observed rate constant of the burst phase. Bar graph values were derived from triplicate experiments and expressed as mean ± standard deviation. Nucleic acid duplexes were denatured by heating at 95°C for 5 min as a positive control and the reaction without protein as a negative control. A large excess of trap RNA (unlabeled top strand) was added to prevent the reannealing of unwound fractions during the reaction.

### 2.5 Annealing assays of complementary single strands

In this study, two partially complementary single strands were used to detect the annealing activity of IBV helicase Nsp13. The strand RNA1 was labeled with FAM at the 5′ end, and the strand RNA2 was not fluorescently labeled. A measure of 4 nM RNA1 was incubated in the 200 μL of reaction buffer (30 mM Tris–HCl, 0.1 mg/mL BSA, 1% glycerol, 3 mM MgCl_2_, and 2 mM DTT) at 37°C with 160 nM NSP13, and the reaction was initiated by adding 4 nM RNA2. Then, 15 μL aliquots were removed at indicated time periods, and terminated the annealing reaction by adding 15 μL of stop buffer containing 50 mM EDTA Na_2_, 1% SDS, and 10% glycerol. A 30 μL of aliquot of each reaction was loaded onto a 12% native polyacrylamide gel and then electrophoresed. The products were visualized by the Typhoon RGB multifunctional laser scanner, and the gray value of the bands was analyzed by ImageJ software. The values in the bar graphs were obtained from three independent measurements, and the data are represented as mean ± standard deviation.

## 3 Results

### 3.1 Expression, purification, and identification of IBV helicase Nsp13

**Figure 1:**
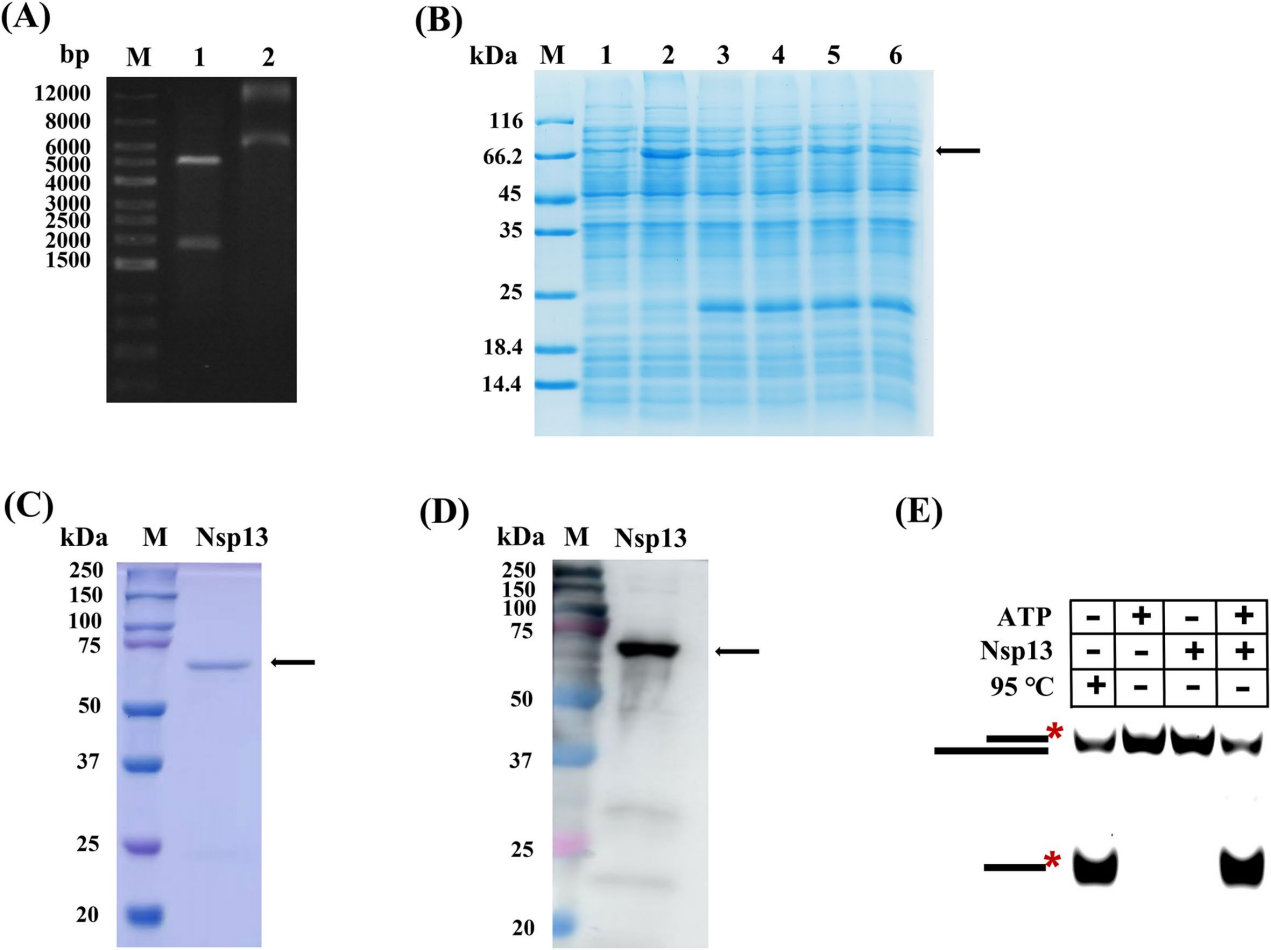
Expression and identification of IBV Nsp13. **(A)** Recombinant plasmid pET-28a-Nsp13. Lane 1: plasmid was digested by double restriction enzymes, lane 2: recombinant plasmid. **(B)** Induced expression of protein Nsp13. Lane 1: uninduced bacterial fluid, lanes 2–6: bacterial fluid after induction. **(C)** Recombinant IBV Nsp13 was analyzed by electrophoresis on a 10% SDS-PAGE gel and stained with Coomassie brilliant blue. **(D)** Nsp13 was analyzed by Western blotting with anti-His antibody. **(E)** Validation of Nsp13 activity by the unwinding reaction. The arrows and asterisks in the figures indicate the target proteins NSP13 and 5′-FAM, respectively.

In our study, the gene *Nsp13* was inserted into the vector pET-28a to construct the recombinant plasmid pET-28a-Nsp13 and then identified by restriction enzyme digestion and sequencing. The positions of the two bands shown in lane 1 were basically consistent with IBV Nsp13 (1800 bp) and pET-28a (5,369 bp) (Figure 1A), which indicated that the recombinant plasmid was successfully constructed. The recombinant plasmid was transformed into BL21 competent cells for protein expression, and lanes 2 to 6 in Figure 1B demonstrated that Nsp13 was successfully expressed. The expanded culture was collected for sonication and fragmentation. Then, the protein was purified by nickel column affinity chromatography and analyzed by SDS-PAGE (Figure 1C) and Western blotting (Figure 1D), and the results indicated that the purified protein was the target helicase Nsp13 (67 kDa). We detected the activity of Nsp13 by the unwinding reaction of duplex substrates, and the products resolved on native PAGE showed that purified Nsp13 exhibited good activity for unwinding dsRNA in the presence of ATP (Figure 1E). In addition, dsDNA was also used as the substrate in the unwinding reaction, and we found that Nsp13 was capable of unwinding both dsDNA and dsRNA in an ATP-dependent manner (Supplementary Figure S1), and Nsp13 exhibited different properties in the unwinding of dsDNA than that in dsRNA and had a clear unwinding preference for dsDNA (Supplementary Figure S2).

### 3.2 Measurement of the ATPase activity of IBV Nsp13

**Figure 2:**
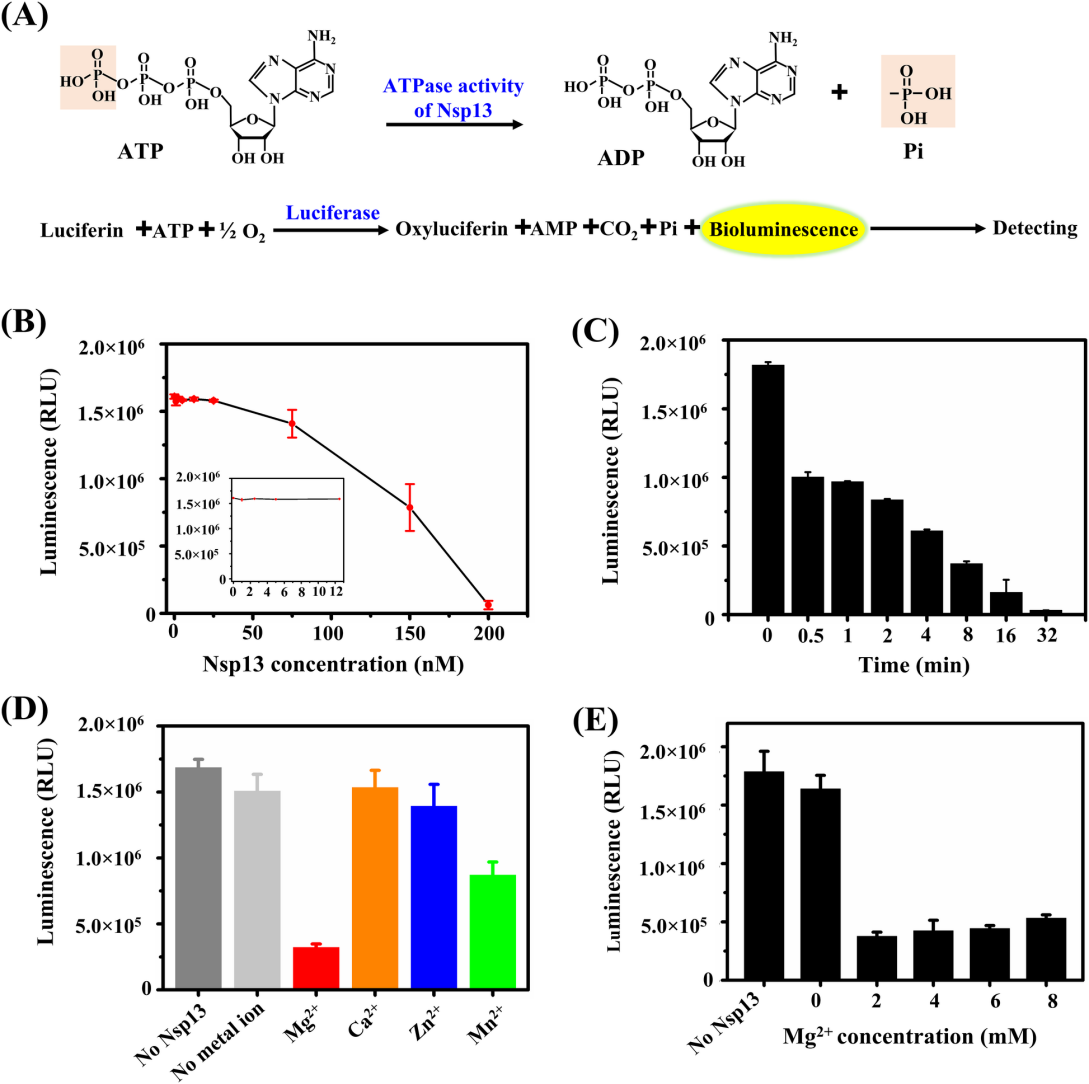
Detection of the ATPase activity of IBV Nsp13. **(A)** Schematic diagram of the principle of the ATPase assay. **(B)** Determination of the ATPase activity at different concentrations of Nsp13. Purified Nsp13 (0, 1, 2.5, 5, 12.5, 25, 75, 150, and 200 nM) were incubated with 5 μM ATP in reaction buffer and then added kinase-lumi reagent, and the bioluminescence was measured. **(C)** The ATPase activity of Nsp13 at the appointed time (0, 0.5, 1, 2, 4, 8, 16, and 32 min) was measured. **(D)** Determination of the ATPase activity of Nsp13 in the presence of 3 mM MgCl_2_, CaCl_2_, ZnCl_2_, and MnCl_2_. **(E)** Determination of the ATPase activity of Nsp13 in the presence of various concentrations of MgCl_2_ (0, 2, 4, 6, and 8 mM). Error bars in figures represent the standard deviation.

According to the results shown in Figure 1E, IBV Nsp13 had an ability to disrupt dsRNA, and the energy from ATP hydrolysis was necessary for the unwinding processes. Moreover, ATP is a major energy resource in cells. Here, we used the kinase-lumi luminescent kinase assay kit to detect the ATPase activity of IBV Nsp13. Luciferase catalyzes the oxidation of luciferin to oxyluciferin in the presence of ATP, and the bioluminescence released during the oxidation of luciferin can be measured to determine the remaining ATP in the reaction mixture (Figure 2A). The luminescence decreased with decreasing the remaining ATP, and the luminescent signal indicated the ATPase activity inversely.

We found that the amount of ATP remaining in the reaction mixture was almost constant when Nsp13 concentrations were in the lower range (from 0 to 12.5 nM), but as the concentration of IBV Nsp13 was increased (from 25 to 200 nM), the amount of ATP decreased dramatically (Figure 2B). In addition, ATP decreased immediately after the addition of 200 nM Nsp13 and reached its lowest level at 32 min, suggesting that IBV Nsp13 rapidly hydrolyzed ATP in a time-dependent manner (Figure 2C).

To explore the requirement of metal ions for ATPase activity of IBV Nsp13, 200 nM Nsp13 was incubated with 5 μM ATP in the presence of 3 mM of MgCl_2_, CaCl_2_, ZnCl_2_, and MnCl_2_. The results showed that Nsp13 possessed optimal ATPase activity in the presence of MgCl_2_, and MnCl_2_ slightly promoted ATPase hydrolysis, and the amount of ATP was virtually unchanged with CaCl_2_ or ZnCl_2_ (Figure 2D). Furthermore, to evaluate the effect of MgCl_2_ concentrations on ATPase activity, Nsp13 was incubated with various concentrations of MgCl_2_. We found that the amount of ATP was significantly reduced by the addition of MgCl_2_ at various concentrations, but the ATPase activity of Nsp13 could be slightly inhibited while the MgCl_2_ concentration was more than 4 mM (Figure 2E). These data demonstrated that Mg^2+^ was critical for the ATPase activity of IBV Nsp13.

### 3.3 Helicase Nsp13 unwinds both dsDNA and dsRNA in a unidirectional manner

**Figure 3:**
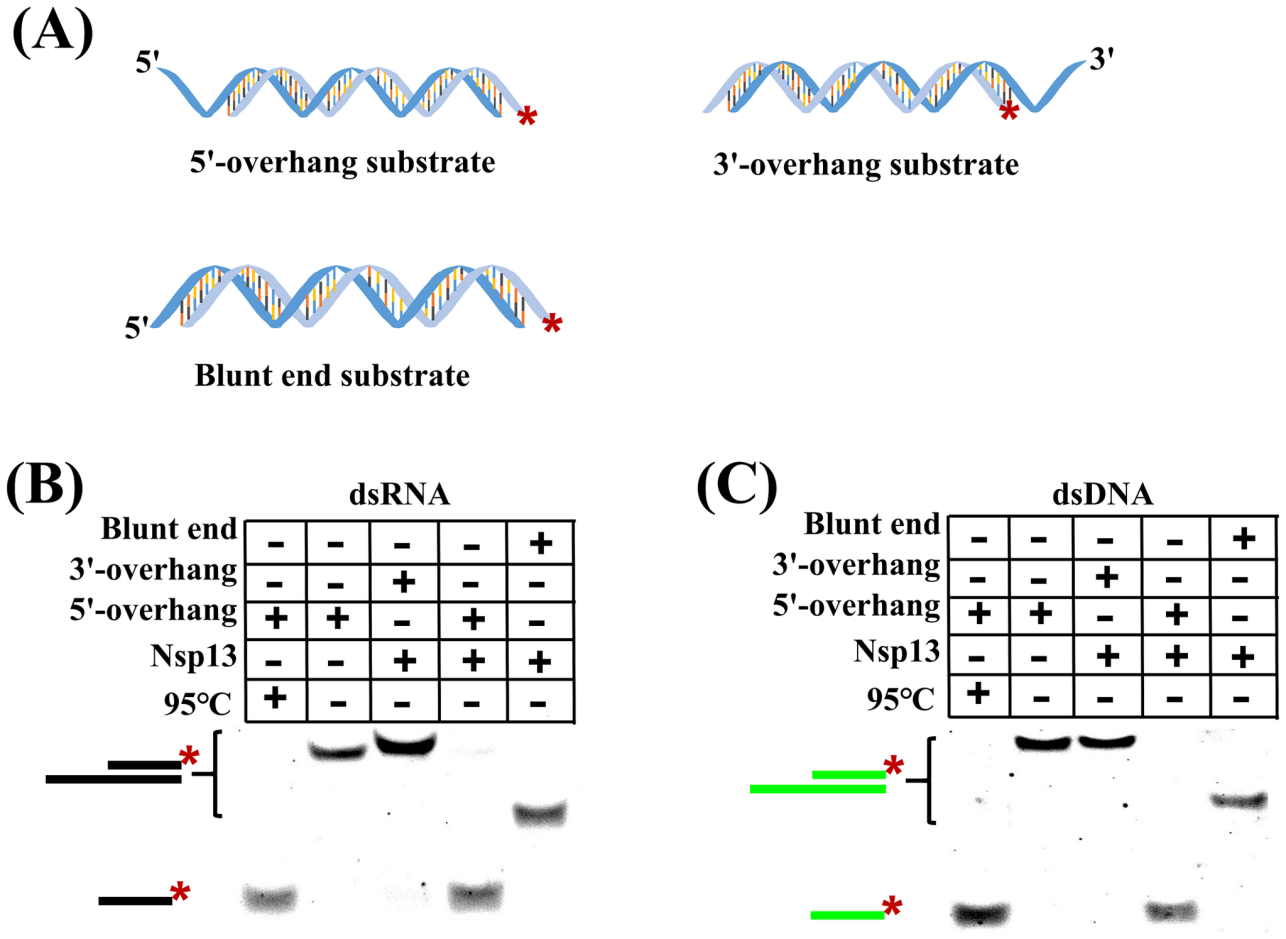
Unwinding polarity of IBV helicase Nsp13. **(A)** Structures of duplex substrates with 5′-overhang, 3′-overhang, and blunt end. **(B)** dsRNA with different structures was unwound by Nsp13. **(C)** dsDNA with different structures was unwound by Nsp13. The asterisk in figures denotes the fluorophore 5′-FAM.

Helicases usually have two distinct pathways to unwind duplex substrates, i.e., directional translocation and local strand separation, but the unwinding fashion of IBV Nsp13 remained unknown. To answer this question, substrates with different secondary structures were designed and used in the unwinding reaction (Figure 3A). We found that IBV helicase Nsp13 was able to unwind dsRNA containing the 5′-overhang but not dsRNA containing the 3′-overhang or blunt end (Figure 3B), and the same results were obtained from the unwinding of dsDNA by Nsp13 (Figure 3C). The unwinding results showed that IBV Nsp13 was a unidirectional helicase with a 5′-to-3′ unwinding polarity and suggested that IBV helicase Nsp13 promoted unwinding initiation by binding to the single-stranded overhang rather than directly to the double-stranded region.

### 3.4 Analysis of the factors that regulate the unwinding activity of Nsp13

**Figure 4:**
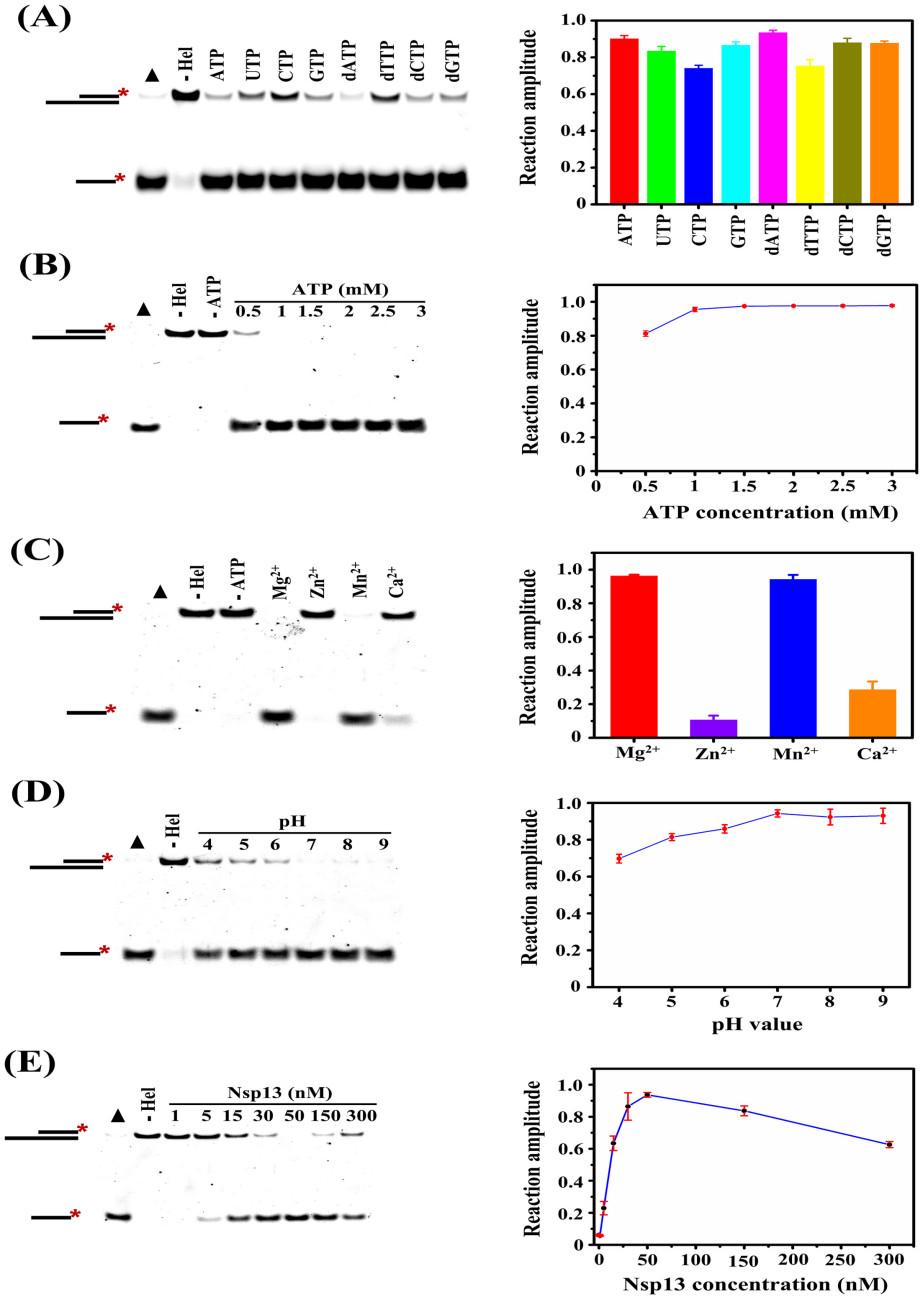
Determination of the factors that regulate the unwinding activity of IBV helicase Nsp13. **(A)** Assessment of the effect of NTP species on the unwinding activity of Nsp13. **(B)** Assessment of the effect of ATP concentration on the unwinding activity of Nsp13. **(C)** Assessment of the effect of metal ions on the unwinding activity of Nsp13. **(D)** Assessment of the effect of pH on the unwinding activity of Nsp13. **(E)** Assessment of the effect of helicase concentration on the unwinding activity of Nsp13. Filled triangles represent the positive controls with heat-denatured substrates, and the unwinding reactions without Nsp13 were the negative controls. The asterisks in the figures indicate the 5′-FAM. The error bars in the figures represent the standard deviation.

Enzyme-catalyzed reactions are affected by a variety of factors, and we explored the effects of NTP species, ATP concentrations, metal ions, solution pH, and helicase concentrations on the unwinding activity of Nsp13 in this study. We found that both NTP and dNTP could be used in the unwinding reaction, but the unwinding efficiency was higher in the presence of ATP and dATP and lower in the presence of CTP and dTTP (Figure 4A). ATP is the major energy source in organisms, and Nsp13 needs to use the energy generated by ATP hydrolysis to translocate and unwind on the substrate nucleic acid strand. In this study, we explored the effects of different concentrations of ATP on the unwinding reaction, and the results showed that the unwinding amplitude reached a maximum at 1 mM ATP (Figure 4B). The results indicated that a small amount of ATP could satisfy the demand of the unwinding reaction.

Metal ions are one of the cofactors of enzymes and act as activators to stimulate the activity, and the species of divalent metal ions affect coronavirus helicase activities (13, 14). The effects of metal ions on the unwinding reaction catalyzed by IBV helicase Nsp13 were investigated in this study, and we found that the unwinding amplitude of substrates reached *A*_(Mg)_ = 0.96 ± 0.01 and *A*_(Mn)_ = 0.94 ± 0.02 in the presence of MgCl_2_ and MnCl_2_, respectively. In contrast, a weak unwinding reaction was observed in the presence of CaCl_2_, and it was almost impossible in the presence of ZnCl_2_ (Figure 4C). Enzyme activity is usually influenced by the pH of the reaction solution, with the highest activity at the optimum pH. In this study, we detected the unwinding reaction catalyzed by IBV Nsp13 in the solution system with different pH. The results showed that the unwinding activity of Nsp13 was lower under acidic conditions, and weakly alkaline conditions were more favorable for the unwinding reaction (Figure 4D).

To explore the effect of helicase concentrations on the unwinding reaction, we carried out the unwinding experiments with various concentrations of Nsp13. Here, the results showed that the unwinding amplitude gradually increased with the increase in Nsp13 concentration, and the unwinding amplitude of 50 nM Nsp13 was *A*_(50 nM)_ = 0.94 ± 0.01. However, the unwinding amplitude appeared to decrease at 150 nM Nsp13 and significantly reduced to *A*_(300 nM)_ = 0.63 ± 0.02 when the enzyme concentration was 300 nM (Figure 4E). The results demonstrated that excessive concentrations of Nsp13, on the contrary, inhibited the unwinding activity of Nsp13.

### 3.5 Nucleic acid continuity of the loading strand is critical for the unwinding reaction catalyzed by Nsp13

**Figure 5:**
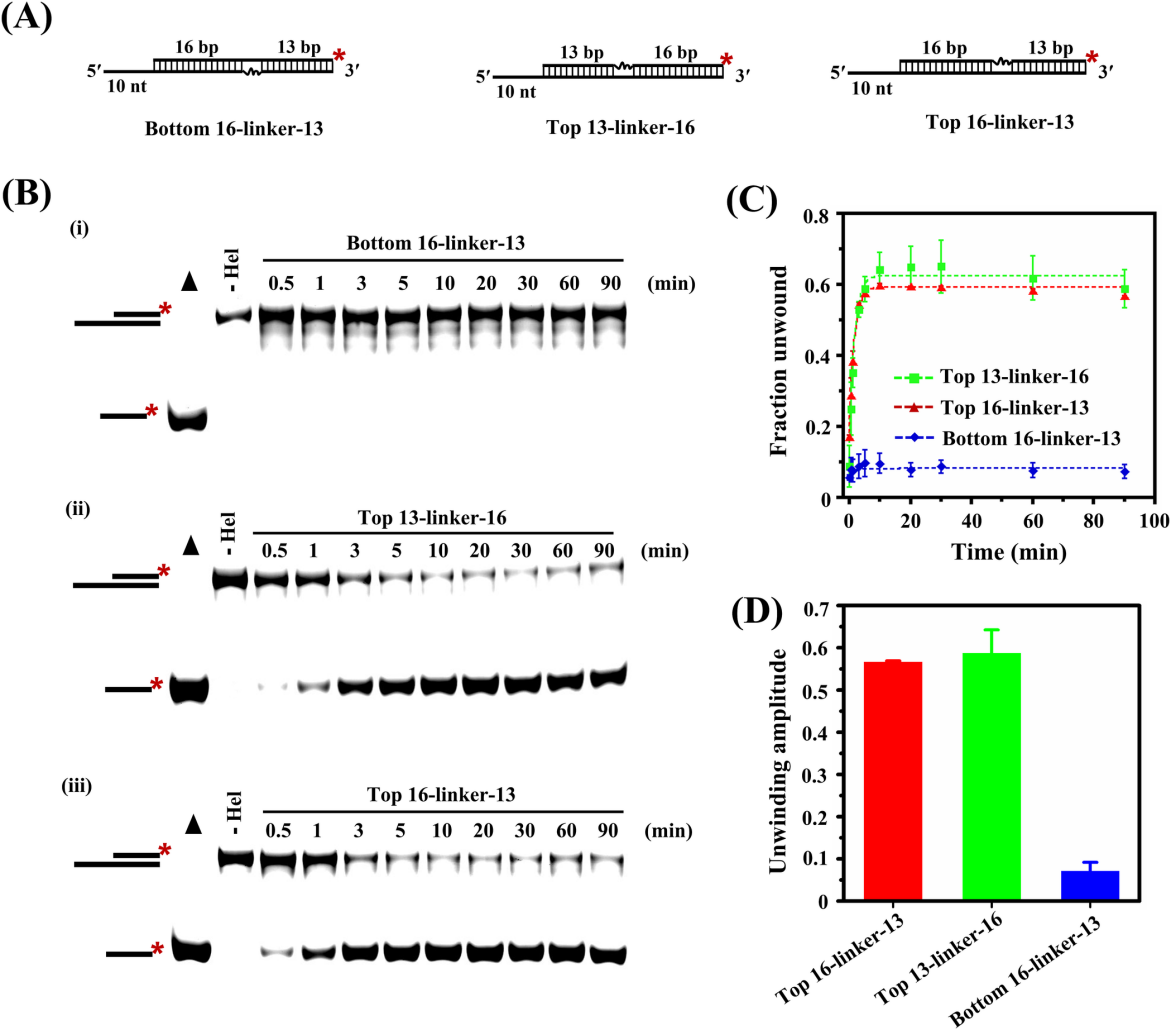
Effect of polyglycol modification of the substrate backbone on the Nsp13-catalyzed unwinding reaction. **(A)** Substrates containing the PEG linker in the top or bottom strand were unwound by Nsp13. **(B)** The reaction products were separated on native PAGE gels. Filled triangles represent the positive controls with heat-denatured substrates, and the unwinding reactions without Nsp13 were the negative controls. **(C)** The derived fractions of RNA unwound at indicated time points were plotted against time. **(D)** The unwinding amplitudes of substrates with various polyglycol modifications. The asterisks in the figures indicate the 5′-FAM. Error bars in figures represent the standard deviation.

The above results clearly showed that IBV Nsp13 translocated on the substrates and moved along the loading strand to unwind the helix, but it was not clear whether a continuous strand of nucleic acid was required for the unwinding reaction. We prepared three substrates that contained 18-atom polyglycol linkers in the top or bottom strand (that was the loading strand); each linker spanned three bases across in the duplex region (Figure 5A). Structural modification of the phosphodiester backbone by the polyglycol linkers disrupted nucleic acid continuity without physically breaking the covalent continuity of the backbone.

Here, we observed strong inhibition of the unwinding activity of Nsp13 when the polyethylene glycol (PEG) linker was positioned in the bottom strand [Figure 5B(i)]. The results indicated that structural modification of the phosphodiester backbone in the bottom strand of the duplex substrates had a significant effect on the unwinding reaction. In addition, the portion of the duplex preceding the linker might be unwinding by Nsp13 but partial unwinding events were undetectable by the methods used here.

To further explore the positional effects of the linker on the unwinding efficiency of Nsp13, the linker was placed at sites closer or farther from the junction, which indicated the joint of the single-stranded region and the paired region (Figure 5B ii and iii). The bands from unwinding reaction were observed in native PAGE, and the results showed that substrates containing a linker in the top strand were readily unwound by Nsp13, the distances of the linkers from the junction had modest effect on the reaction amplitudes, and the effect on the reaction rates was negligible (Figures 5C,D). In our current study, the results provided biochemical evidence that the nucleic acid continuity of the bottom strand was indispensable for the unwinding reaction catalyzed by IBV Nsp13.

### 3.6 Discovery of the annealing activity of IBV helicase Nsp13

Our study demonstrated that excess concentrations of Nsp13 inhibited the unwinding of duplex substrates, but it was not clear whether Nsp13 had an opposite function to unwinding, i.e., Nsp13 promoted the annealing of two complementary single strands to form a double strand.

**Figure 6:**
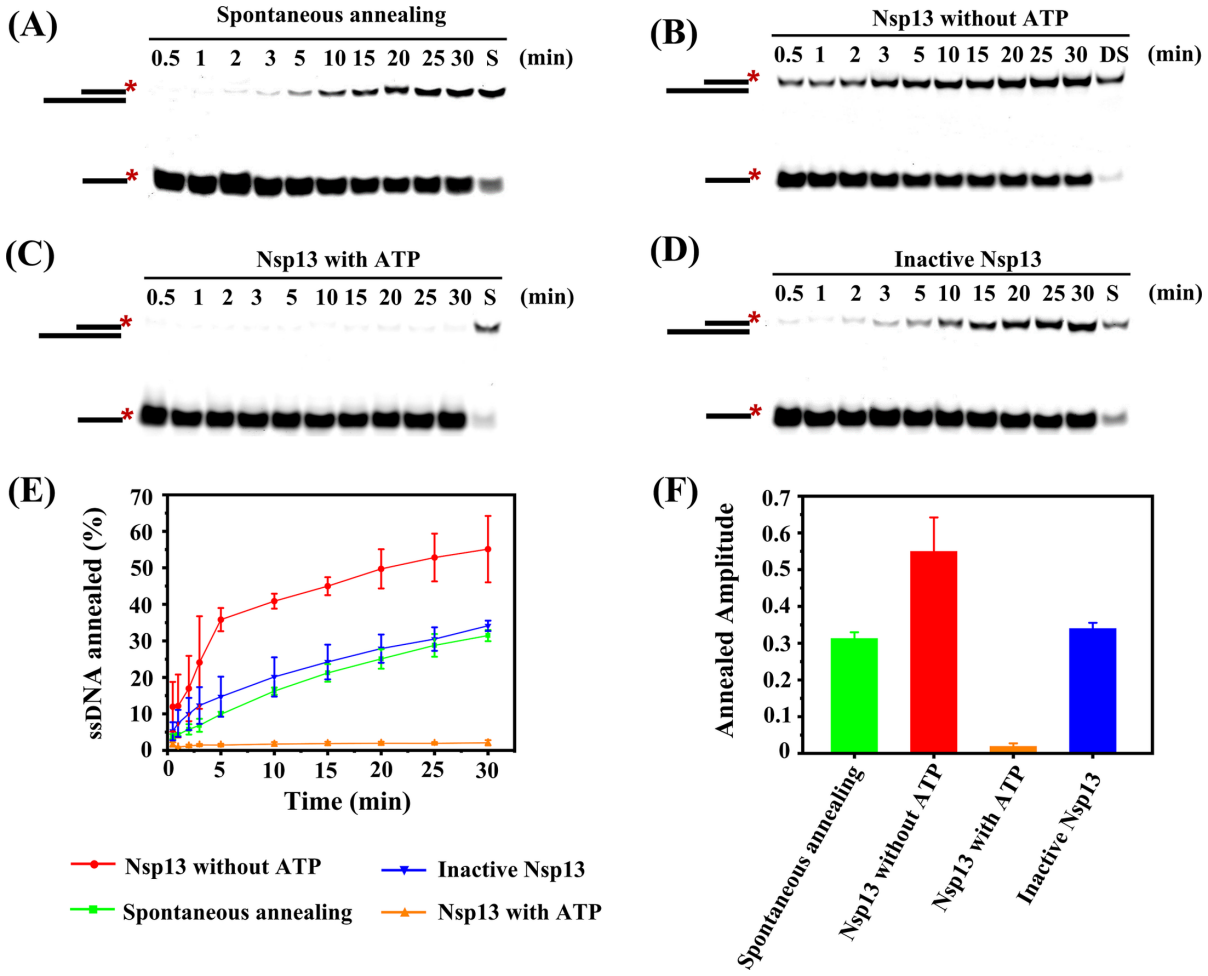
Determination of the annealing activity of IBV Nsp13. **(A)** Spontaneous annealing of ssRNA was detected in the reaction. **(B)** Annealing reaction of single-stranded RNA was catalyzed by Nsp13 in the absence of ATP. **(C)** Annealing reaction of single-stranded RNA was catalyzed by Nsp13 in the presence of ATP. **(D)** Annealing reaction of single-stranded RNA was catalyzed by inactive Nsp13. **(E)** The percentage of dsRNA formed in the annealing reaction was plotted against time to show the progress of product formation. **(F)** The amplitudes of the annealing reaction under various conditions. The asterisks in the figures indicate the 5′-FAM. Error bars in figures represent the standard deviation.

In this study, we observed the annealing reaction under different conditions, and we found that two single strands spontaneously annealed to form double strands without Nsp13 (Figure 6A) or in the presence of inactive Nsp13 (Figure 6D). Here, Nsp13 was denatured at 95°C for 10 min to be inactivated (Supplementary Figure S3). In contrast, the annealing efficiency was significantly enhanced by adding Nsp13 in the absence of ATP (Figure 6B), but the annealing phenomenon was almost invisible by simultaneously adding ATP and Nsp13 (Figure 6C). The results showed that Nsp13 facilitated the annealing of ssRNA to yield dsRNA with an efficiency of 55% in the absence of ATP, whereas spontaneous annealing of RNA without Nsp13 or with inactive Nsp13 produced only approximately 30% dsRNA. The annealing reaction was completely inhibited by 3 mM ATP, and no dsRNA was produced (Figures 6E,F). The results indicated that IBV Nsp13 displayed ATP-independent annealing activity.

## 4 Discussion

Coronaviruses can affect humans and a wide variety of animal species, and the spread of animal coronaviruses impairs public health security and economic development (16). Diseases caused by IBV in chickens lead to reduced growth performance and egg production, resulting in serious economic losses for the chicken industry, and the development of effective antiviral drugs has become one of the most important treatments for virus infection (17–19). The helicase Nsp13 encoded by the IBV genome is an ideal drug target; thus, it is necessary to study the biochemical properties of Nsp13. In this study, IBV Nsp13 was expressed and purified for unwinding assays, and various duplexes were designed as substrates. The results demonstrated that Nsp13 was able to disrupt the helix of substrates with a 5′-overhang in an ATP-dependent manner, regardless of whether the substrate was DNA or RNA, similar to other coronavirus helicases (12, 20).

To unwind duplexes, helicases typically load onto one of the two nucleic acid strands, usually in a single-stranded region, and then translocate on this strand in a unidirectional fashion, thereby separating the complementary DNA or RNA (21). In this study, IBV Nsp13 promotes unwinding initiation by binding to the 5′-overhang in a classic ‘directional translocation’ mode, while the DEAD-box protein Ded1 directly binds to the double-stranded region and unwinds duplex substrates by a ‘local strand separation’ mode (22, 23).

IBV helicase Nsp13 preferred to use Mg^2+^ and Mn^2+^ for optimal helix unwinding. Moreover, Nsp13 could utilize the energy from all NTP hydrolysis to unwind duplex substrates, suggesting that the nucleotide-binding site of IBV Nsp13 has a low specificity among NTPs. Similar data were also obtained for SARS-CoV Nsp13 (24). Due to high nucleotide consumption was happened in viral RNA synthesis in the host cell, it may be beneficial for the unwinding activity of helicase was not strictly dependent on specific nucleotide, reduced the risk of depleting cellular NTP pools (13). As known, enzyme concentration is the key factor affecting the results of the enzymatic reaction. Here, we found that the unwinding efficiency was not always increased with increasing helicase concentrations, and excess concentrations of Nsp13 inhibited the unwinding reaction. This finding could contribute to the fact that too many helicase molecules wrapped around the substrate to form a relatively stable helicase–substrate complex, which ultimately affected the movement of the helicase on the substrate and led to a decrease in the unwinding efficiency.

In our study, the unwinding reaction of duplex substrates with structural modification was tested, and each substrate contained a PEG linker that spanned three bases and replaced the sugar-phosphate backbone. We found that the unwinding activity of Nsp13 required the nucleic acid continuity of the loading strand of duplex substrates. Previous studies have also reported that the nucleic acid continuity of the single-stranded tail was necessary for some helicases to effectively load and unwind duplex substrates (25–27). However, helicase Rep of *E. coli* had a unique mechanism that tolerated backbone disruption for loading and unwinding of duplex substrates (28).

**Figure 7:**
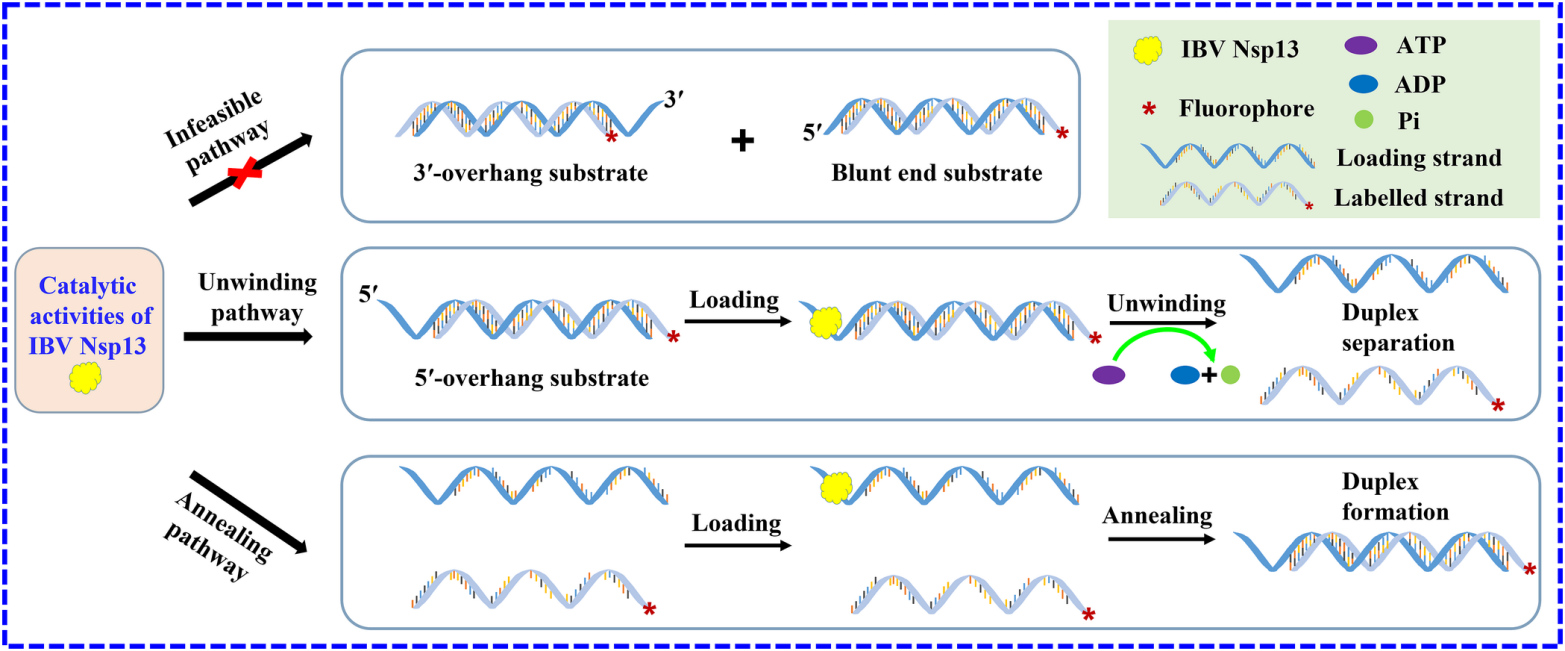
Scheme of the catalytic activities of IBV helicase Nsp13. Nsp13 unwinds duplex substrates with 5′-overhang by utilizing the energy derived from ATP hydrolysis and promotes the annealing of complementary single strands in the absence of ATP. The asterisks in the figures indicate the 5′-FAM.

There are several helicases that not only have unwinding activity but also catalyze the annealing of single strands, such as DEAD-box helicase CsdA, Ded1, and Mss116 (29–31). Here, the evidence first demonstrated that IBV helicase Nsp13 also possessed rewinding activity; in other words, Nsp13 was able to significantly promote the annealing of ssRNA to generate dsRNA in the absence of ATP. The annealing activity of IBV Nsp13 in our study was an extremely important finding and filled the knowledge gap of the annealing function of coronavirus helicases. The unwinding of the double strands and annealing of the single strands might have existed concurrently during the separating process of duplex substrates, but the annealing phenomenon was masked because of the higher unwinding efficiency in the presence of ATP (Figure 7). As a result, IBV Nsp13 had abilities to promote both duplex unwinding and strand annealing, and it implied that Nsp13 might be able to facilitate more complex RNA structural rearrangements than simple duplex unwinding or helix formation. Therefore, we speculated that Nsp13 also possessed the RNA chaperone activity. Nsp13 has five domains with profound effects on helicase function and viral propagation (32–34). We have now expressed the truncated protein *in vitro* and carried out a series of unwinding experiments to explore the effect of different domains on Nsp13 activity. However, the specific functions of the various domains of IBV Nsp13 are not fully understood and deserve further study.

## Data Availability

The original contributions presented in the study are included in the article/Supplementary material, further inquiries can be directed to the corresponding author.
