# Supplementary material for: Two opposite abilities of the infectious bronchitis virus helicase Nsp13: separating the duplex and promoting the annealing of single-stranded nucleic acid
Source: Front Vet Sci. 2025 Mar 17;12:1560586. doi: 10.3389/fvets.2025.1560586 (PMC11955809; doi:10.3389/fvets.2025.1560586)
Supplement: Supplementary file 1 [file Table_1.DOCX]

Supplementary Material

**Table S1.** Single-stranded RNA or DNA used in this study

| **Names Sequences (5′→3′)** |
| --- |
| RNA1***** FAM-GCGUCGUAUCGAUCU |
| RNA2 CAGCUAGACCAGAUCGAUACGACGC  RNA3 AGAUCGAUACGACGCCCAGAUCGAC  RNA4 AGAUCGAUACGACGC  RNA5***** FAM-GCGUCGUAUGCUGACUCGAUCCUGACGGUGAC  RNA6 CAGUGGCAGUCCUAGCUCAGUCGUAUGCUGCG-***linker***-CAGCAUACGACGC  RNA7 CUAGACCAGAGUCACCGUCAGGAUCGAGUCAGCAUACGACGC  RNA8***** FAM-GCGUCGUAUGCUGACU-***linker***-UCCUGACGGUGAC  RNA9***** FAM-GCGUCGUAUGCUG-***linker***-CGAUCCUGACGGUGAC  DNA1***** FAM-GCGACGTCACGTGCA  DNA2 CAGCTAGACCTGCACGTGACGTCGC  DNA3 TGCACGTGACGTCGCCCAGATCGAC  DNA4 TGCACGTGACGTCGC |

Note: asterisks in tables denote the fluorescent dye FAM.


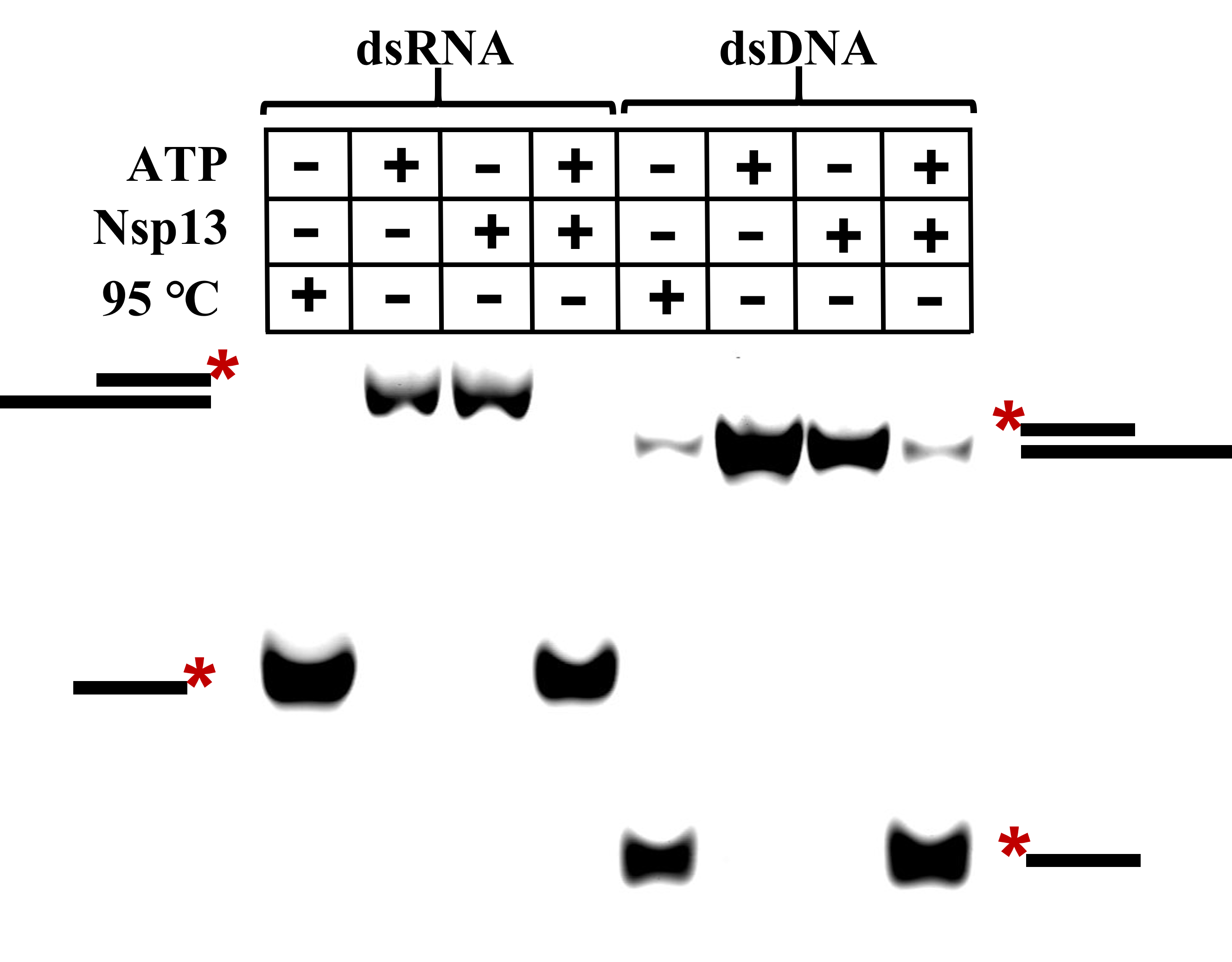


**Figure S1.** IBV helicase Nsp13 unwinds dsRNA and dsDNA in ATP-dependent manner. Both dsRNA and dsDNA were unwound by using the energy from ATP hydrolysis, and substrates remained duplex structure without ATP. Duplex substrates were denatured at 95 ℃ as a positive control, and the reaction without Nsp13 as a negative control. The red asterisk represents the FAM used for labeling the substrates.


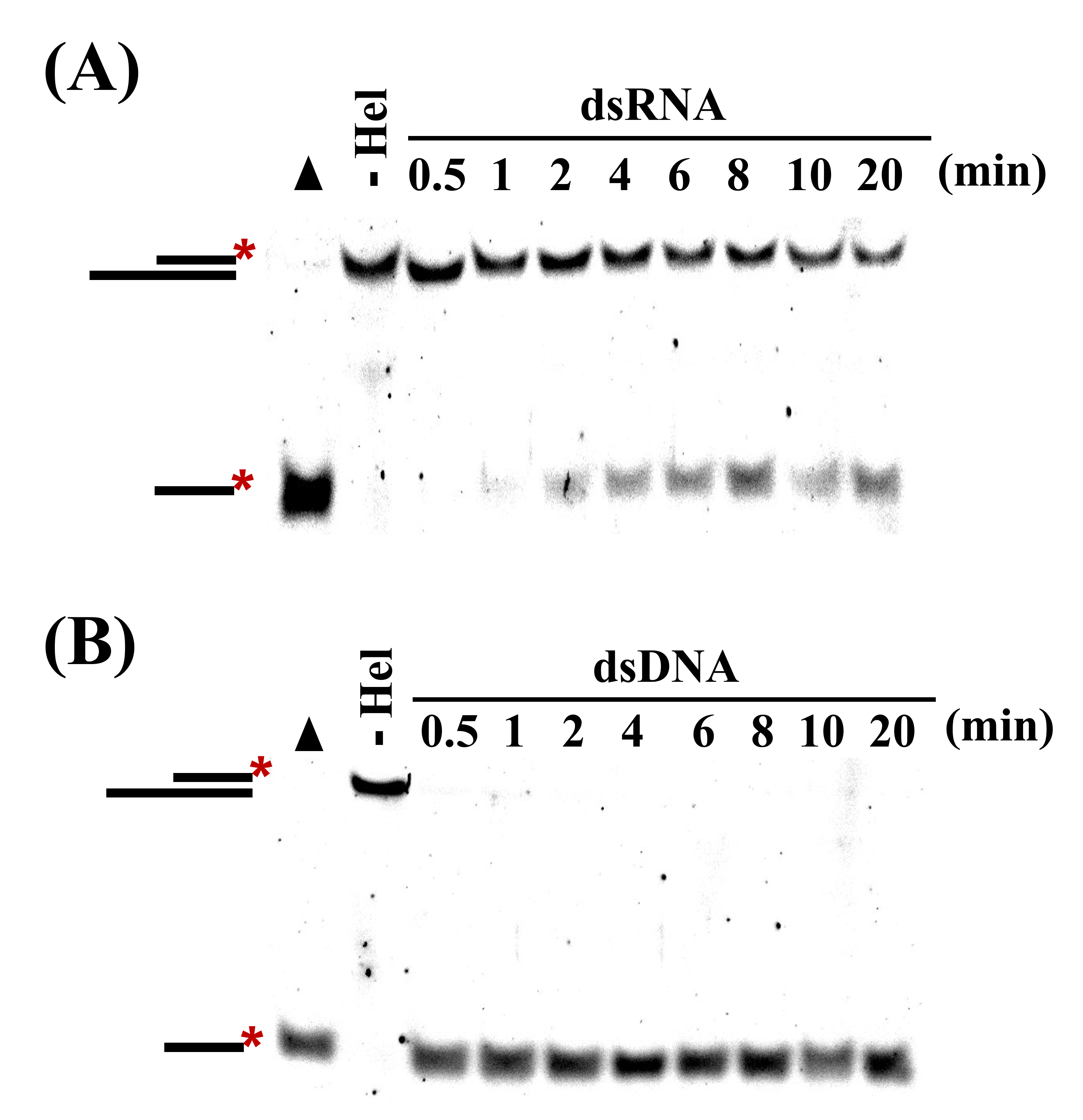


**Figure S2.** Substrate preference of the unwinding activity of Nsp13. **(A)** Unwinding products of dsRNA by Nsp13 for indicated time points. **(B)** Unwinding products of dsDNA by Nsp13 for indicated time points. The asterisks in the figures indicate the 5ʹ-FAM.


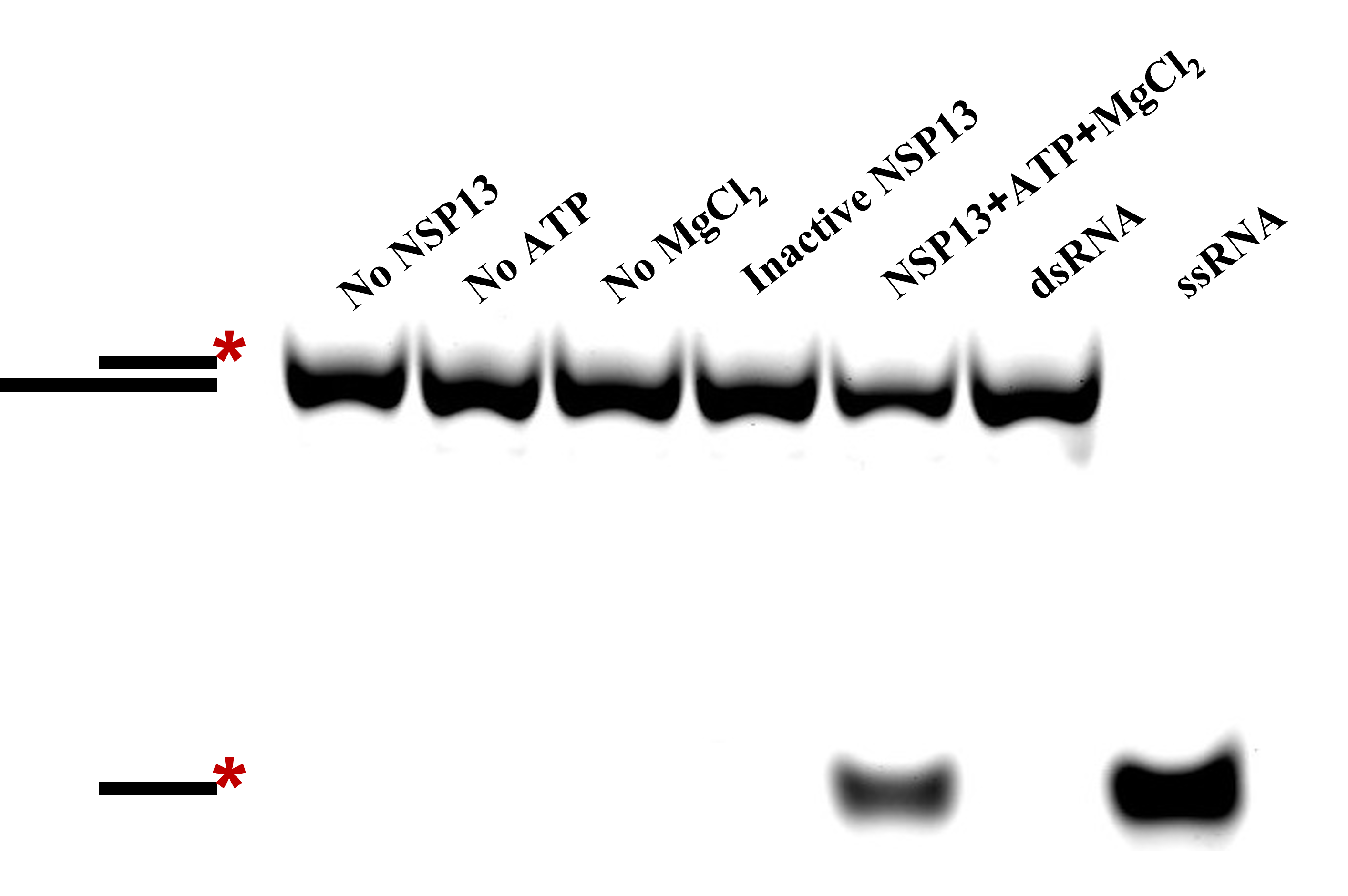


**Figure S3.** IBV Nsp13-catalyzed unwinding reaction in various conditions. Duplex substrates were not unwound in the absence of Nsp13 or ATP or MgCl_2_ or inactive Nsp13, and IBV helicase Nsp13 had ability to unwind duplex substrates in the presence of ATP and MgCl_2_. Nsp13 was denatured at 95 ℃ for 10 min to be inactivated, dsRNA and ssRNA were negative control and positive control, respectively. The asterisks in the figures indicate the 5ʹ-FAM.
